# Allelic Entropy and Times until Loss or Fixation of Neutral Polymorphisms

**DOI:** 10.3390/genes14091751

**Published:** 2023-09-02

**Authors:** Luca Ferretti, Paolo Ribeca

**Affiliations:** 1 Pandemic Sciences Institute and Big Data Institute, Nuffield Department of Medicine, University of Oxford, Oxford OX3 7LF, UK; 2 Biomathematics and Statistics Scotland, The James Hutton Institute, Edinburgh EH9 3FD, UK; paolo.ribeca@hutton.ac.uk

**Keywords:** diffusion approximation, fixation time, fixation probability, genetic drift, superlinear population growth, allelic entropy, neutral variants

## Abstract

In the diffusion approximation of the neutral Wright–Fisher model, the expected time until fixation or loss of a neutral allele is proportional to the initial entropy of the distribution of the allele in the population. No explanation is known for this coincidence. In this paper, we show that the rate of entropy dissipation is proportional to the number of segregating alleles. Since the final fixed state has zero entropy, the expected lifetime of segregating alleles is proportional to the initial entropy in the system. We show that classical formulae on the average time to loss of segregating alleles and the expected time to fixation of the last segregating allele stem from these properties of the diffusion process. We also extend our results to the case of population size changing in time. The dissipation of heterozygosity and entropy shows that superlinear population growth leads to infinite expected fixation times, i.e., neutral alleles in fast-growing populations could segregate forever without ever becoming fixed or disappearing by genetic drift.

## 1. Introduction

Fixation times of variants in natural populations have been an important object of study since the birth of modern population genetics (see Ewens [1] and citations therein). They have been widely studied in standard discrete-time neutral models describing the evolution of neutral alleles under genetic drift, such as the Wright–Fisher [2] and Moran models [1], especially in their diffusion approximation [3]. A classic result for the expected time $t_{FL}$ until fixation or loss of a neutral allele, derived by the diffusion approximation of the Wright–Fisher model, is

(1) $$E\left[t_{FL}\right]\propto p\ln p+\left(1-p\right)\ln(1-p)$$

where *p* is the initial frequency of the allele. A striking observation is that the quantity that appears on the right side corresponds to the mathematical form of an entropy. It could be the Shannon entropy associated with the Bernoulli sampling of an individual with or without that allele from the population, or the Boltzmann entropy of the system in terms of the number of microstates compatible with a macroscopic frequency *p* for that allele, since the two are closely related [4].

This formal correspondence between Equation (1), i.e., time until loss or fixation of a neutral allele, and entropy was noticed *en passant* by Buss and Clote [5], who proved Equation (1) in a weaker form for a more general class of models with finite population size. However, no simple explanation is known for this coincidence.

In this paper, we discuss a clear connection between the allelic entropy of variants in a neutral population and the time until loss or fixation of the alleles. As a consequence of the diffusion dynamics of neutral alleles and the definition of entropy, the average rate of entropy dissipation is proportional to the number of segregating alleles minus one. Since the entropy of the final state with a single fixed allele is zero, the overall change in entropy should be equal to the initial one. This is the basis of the relation between the lifetime of segregating alleles and the initial entropy. We use this approach to (re)derive formulae for the average time until loss of a segregating allele in a multiallelic population, as well as the expected time to fixation of the last surviving allele.

In the last sections, we tackle the case of nonconstant population size N(t). We prove that there are two different cases, depending if the $\int_{0}^{\infty }dt/N\left(t\right)$ is infinite or finite. In the first case, i.e., for populations with sublinear or linear growth, all polymorphisms get fixed or extinct in the long term, and there is still a correspondence between the initial entropy and the expectation values of nonlinear rescalings of times until fixation/loss. In the second case, i.e., for exponential growth or other forms of superlinear growth, the expected times to fixation or loss are infinite. This corresponds to alleles segregating forever in the population with a nonzero probability.

## 2. Materials and Methods

### 2.1. Entropy Dissipation and Times to Fixation

Throughout this paper, we consider a population of large size *N* evolving according to the diffusion approximation of the neutral Wright–Fisher model without mutations.

Assume that the population is polymorphic with *A* possible types (alleles). Let $k_{1},k_{2}\ldots k_{A}$ be the counts of the different alleles in the population (with $\sum_{i=1}^{A}k_{i}=N$) and $f_{1},f_{2}\ldots f_{A}$ the corresponding frequencies, i.e., $f_{i}=k_{i}/N$. Then, the (Boltzmann) entropy of the polymorphism in the population is defined as SBoltzmann=lnNk1,k2…kA. For large *N*, $S_{Boltzmann}\approx S$ defined as

(2) $$S=-N\sum_{i=1}^{A}f_{i}\ln f_{i}$$

This corresponds also to *N* times the Shannon entropy of the sampling distribution of an allele from the population. This entropy is positive definite, and it is zero if and only if the population is monomorphic (i.e., for some *i* we have $f_{i}=1$, $f_{j}=0$ for j≠i).

#### 2.1.1. Biallelic Entropy and Time until Fixation/Loss

The Wright–Fisher process on the frequency *f* of a neutral allele satisfies [1,3]

(3) $$E\left[\Delta f|f\right]=0\;,\;E\left[{\left(\Delta f\right)}^{2}|f\right]=\frac{f(1-f)}{N}\;,\;E\left[{\left(\Delta f\right)}^{k}|f\right]=o\left(\frac{1}{N}\right)\;\mathrm{for}\;k\geq 3$$

where Δf is the variation in frequency in a single generation. The scale and speed of the corresponding one-dimensional diffusion process are *f* and $\frac{N}{f(1-f)}$, respectively [1].

From the Kolmogorov equations, for two alleles of frequency *f* and 1−f, the entropy dissipated in a single generation is

(4) $$E\left[\Delta S|f\right]=\frac{dS}{df}\cdot E\left[\Delta f|f\right]+\frac{1}{2}\frac{d^{2}S}{df^{2}}\cdot E\left[{\left(\Delta f\right)}^{2}|f\right]=\frac{1}{2}\left(-N\frac{\delta_{\left[0<f<1\right]}}{f(1-f)}\right)\cdot \frac{f(1-f)}{N},$$

hence the final result

(5) $$E\left[\Delta S|f\right]=-\frac{\delta_{\left[0<f<1\right]}}{2}$$

where $\delta_{\left[X\right]}$ is the indicator function of *X*, i.e., a function that is 1 if *X* is true and 0 otherwise.

#### 2.1.2. Multiallelic Entropy and Time until Loss

Consider a Wright–Fisher process with $A_{0}$ initial alleles of initial frequencies $f_{1,0},f_{2,0}\ldots$$f_{A_{0},0}$. Denote the number of segregating alleles in time as $A\left(t\right)=\sum_{i=1}^{A_{0}}\delta_{\left[0<f_{i}\left(t\right)<1\right]}$ with the initial condition $A\left(0\right)=A_{0}$.

The diffusion approximation for this system implies [1]

(6) $$E\left[\Delta f_{i}\right]=0\;,\;E\left[\Delta f_{i}\cdot \Delta f_{j}\right]=\frac{f_{i}\delta_{ij}-f_{i}f_{j}}{N}\;,\;E\left[{\left(\Delta f\right)}^{k}\right]=o\left(\frac{1}{N}\right)\;\mathrm{for}\;k\geq 3;$$

therefore, the decrease in entropy in a generation is

(7) $$E\left[\Delta S\right]=\frac{1}{2}\sum_{i,j=1}^{A_{0}}\frac{\partial^{2}S}{\partial f_{i}\partial f_{j}}\cdot E\left[\Delta f_{i}\Delta f_{j}\right]=\frac{1}{2}\sum_{i=1}^{A_{0}}\left(-N\frac{\delta_{\left[0<f_{i}<1\right]}}{f_{i}}\right)\cdot \frac{f_{i}\left(1-f_{i}\right)}{N}=-\sum_{i=1}^{A_{0}}\frac{\delta_{\left[0<f_{i}<1\right]}\left(1-f_{i}\right)}{2},$$

hence

(8) $$E\left[\Delta S\right]=-\frac{A(t)-1}{2}$$

We denote by $j_{1}\ldots j_{A_{0}}$ the allele indices ordered by survival and by $t_{L,j_{1}}<t_{L,j_{2}}\ldots <t_{L,j_{A_{0}}}$ the corresponding lifetimes (both indices and times are random variables). Note that in absence of mutations, the $j_{A_{0}}$th allele gets fixed forever in the population and $t_{L,j_{A_{0}}}=\infty$. By integrating the Equation (27), we obtain

(9) $$\begin{array}{cc} 2S_{0}= & \int_{0}^{\infty }dt\;E\left[\sum_{i=1}^{A_{0}}\delta_{\left[0<f_{i}\left(t\right)<1\right]}-\delta_{\left[0<f_{j_{A_{0}}}\left(t\right)<1\right]}\right]=\sum_{i=1}^{A_{0}-1}\int_{0}^{\infty }dt\;E\left[\delta_{\left[0<f_{j_{i}}\left(t\right)<1\right]}\right]\\ = & \sum_{i=1}^{A_{0}-1}\int_{0}^{\infty }dt\;P\left[t_{L,j_{i}}>t\right]=\sum_{i=1}^{A_{0}-1}\int_{0}^{\infty }dt\;t\;p\left[t_{L,j_{i}}=t\right]=\sum_{i=1}^{A_{0}-1}E\left[t_{L,j_{i}}\right] \end{array}$$

We can rewrite this in terms of the time of loss $t_{L}$ of a random allele among the $A_{0}-1$ alleles not going to fixation. The expected time until loss is $E\left[t_{L}\right]=\frac{1}{A_{0}-1}\sum_{i=1}^{A_{0}-1}E\left[t_{L,j_{i}}\right]$, hence

(10) $$E\left[t_{L}\right]=\frac{2S_{0}}{A_{0}-1}=-\frac{2N}{A_{0}-1}\sum_{i=1}^{A_{0}}f_{i,0}\ln(f_{i,0})$$

#### 2.1.3. Time until Fixation

A more interesting result can be obtained by combining the results above. The sum of all times until loss/fixation of each allele is

(11) $$\sum_{i=1}^{A_{0}}E\left[t_{FL,i}\right]=2S_{0}-2N\sum_{i=1}^{A_{0}}\left(1-f_{i,0}\right)\ln(1-f_{i,0})$$

but the same sum can be decomposed as the sum of the expected time to fixation of the last allele and the sum of times of loss of the others: $\sum_{i=1}^{A_{0}}E\left[t_{FL,i}\right]=E\left[t_{F}\right]+\sum_{i=1}^{A_{0}-1}E\left[t_{L,j_{i}}\right]=E\left[t_{F}\right]+2S_{0}$. The expected time to fixation of the last surviving allele is therefore

(12) $$E\left[t_{F}\right]=-2N\sum_{i=1}^{A_{0}}\left(1-f_{i,0}\right)\ln(1-f_{i,0})$$

For a slightly different point of view on these results, define the quantities $S_{C,i}=-N\left(1-f_{i}\right)\ln(1-f_{i})$. Their expectation values satisfy

(13) $$E\left[\Delta S_{C,i}\right]=-\frac{f_{i}\delta_{\left[0<f_{i}<1\right]}}{2}\;\;\Rightarrow \;\;S_{C,i}\left(0\right)-E\left[S_{C,i}\left(\infty \right)\right]=\frac{1}{2}E\left[\int_{0}^{t_{FL,i}}f_{i}\left(t\right)dt\right]$$

where the last integral can be reinterpreted in terms of the fixation time $t_{F,i}$ and fixation probability $P_{fix,i}=f_{i}$ as follows:

$$\begin{array}{cc} E\left[\int_{0}^{t_{FL,i}}f_{i}\left(t\right)dt\right]= & E\left[\int_{0}^{\infty }\int_{0}^{1}fP\left[f_{i}\left(t\right)=f\right]dfdt\right]\\ = & E\left[\int_{0}^{\infty }\int_{0}^{1^{-}}P_{fix,i|f}\delta_{\left[f<1\right]}P\left[f_{i}\left(t\right)=f\right]df\;dt\right]\\ = & P_{fix,i}E\left[t_{FL,i}|i\;\mathrm{fixed}\right]=P_{fix,i}E\left[t_{F,i}\right]. \end{array}$$

Once we notice that $E[S_{C,i}\left(\infty \right)]=0$, this formula is equivalent to the classical results of Kimura and Ohta [6] on the expected times to fixation $E\left[t_{F,i}\right]=-2N\left(1-f_{i,0}\right)\ln(1-f_{i,0})/f_{i,0}$ for alleles of initial frequency $f_{i}$. Equation (30) can the be found by weighting $E[t_{F,i}]$ by the neutral probabilities of fixation $P_{fix,i}=f_{i,0}$.

From yet another point of view, the sum $S_{C}=\sum_{i=1}^{A}S_{C,i}$ has a constant rate of dissipation per generation, as long as some alleles are still segregating:

(14) $$S_{C}=-N\sum_{i=1}^{A}\left(1-f_{i}\right)\ln(1-f_{i})\;\;\Rightarrow \;\;E\left[\Delta S_{C}\right]=-\frac{\delta_{\left[A(t)>1\right]}}{2}$$

which in turn implies $E\left[t_{F}\right]=2\left(S_{C}\left(0\right)-E\left[S_{C}\left(\infty \right)\right]\right)$, which is equivalent to Equation (30), since fixation or loss occurs in finite time.

### 2.2. Variable Population Size

#### 2.2.1. Entropy and Rescaled Times to Fixation/Loss

Given the population size $N_{0}$ at the initial time, we define the relative population size $\lambda \left(t\right)=N\left(t\right)/N_{0}$. We also define the rescaled entropy as s(t)=S(t)/λ(t) (and $s_{C}\left(t\right)=S_{C}\left(t\right)/\lambda \left(t\right)$). These quantities change only after a change in frequencies, not in population size only.

By the same reasoning as before, we find that the rescaled entropy satisfies the generalized equation

(15) $$E\left[\Delta s|f_{1}\ldots f_{A_{0}}\right]=-\frac{A(t)-1}{2\lambda (t)}$$

If we change variable from *t* to $\tau =\int_{0}^{t}du/\lambda \left(u\right)$, we find that the average decay of rescaled entropy is linear in τ. Note also that s(0)=S(0). Therefore, provided that $\int_{0}^{\infty }dt/\lambda \left(t\right)=+\infty$ (which ensures that all alleles are going to fixation or loss in a finite time, as we discuss in the next section), the formulae of the previous sections retain their validity once each time is replaced by the corresponding rescaled time.

#### 2.2.2. Superlinear Growth and Infinite Fixation Times

For the case of superlinear growth, $\int_{0}^{\infty }dt/\lambda \left(t\right)<+\infty$, and the following theorem applies.

**Theorem** **1.**  *Consider a population with effective size $N\left(t\right)=\lambda \left(t\right)N_{0}$ and a finite number of alleles $A_{0}>1$ at time t=0, evolving according to the diffusion process in Equation (6). Denote the probability that alleles are segregating in the population at time t by P[A(t)>1]. Define $p_{\infty }=h_{0}{\left(1-1/A_{0}\right)}^{-1}e^{-\int_{0}^{\infty }dt/N\left(t\right)}$.*

*(i)*  *If $\tau_{max}=\int_{0}^{\infty }dt/\lambda \left(t\right)=+\infty$, P[A(t)>1] converges to 0 for t→+∞, i.e., there are no segregating alleles at t=+∞.*
*(ii)*  *If $\tau_{max}=\int_{0}^{\infty }dt/\lambda \left(t\right)<+\infty$, P[A(t)>1]→c with $c\geq p_{\infty }$ for t→+∞, i.e., there is a nonzero probability that alleles are still segregating in the population at time t=+∞.*

**Proof.**  First, consider the positive function P[A(t)>1]. For times $t^{\prime \prime }>t^{\prime }$, $A\left(t^{\prime \prime }\right)>1\Rightarrow A\left(t^{\prime }\right)>1$. This implies that P[A(t)>1] is monotonically decreasing in time; hence, it converges to some limit P[A(t)>1]→c≥0 as t→∞.

To prove the statement (i), assume that $\lim_{t\to \infty }P\left[A\left(t\right)>1\right]=c>0$. Since N(t)>0, the rescaled time τ(t) is a strictly increasing function of *t*. Hence, $\lim_{\tau \to \infty }P\left[A\left(t\left(\tau \right)\right)>1\right]=c>0$. This implies $E\left[\tau_{F}\right]=\int_{0}^{\infty }d\tau P\left[A\left(t\left(\tau \right)\right)>1\right]\geq c\int_{0}^{\infty }d\tau =+\infty$. But, using the linear decay in τ and the positivity of the entropy $s_{C}$, we have $E\left[\tau_{F}\right]=S_{C}\left(0\right)-\lim_{t\to \infty }E\left[s_{C}\left(t\right)\right]\leq S_{C}\left(0\right)<+\infty$, i.e., the expected fixation time $E[\tau_{F}]$ is finite; hence, by contradiction, we have c=0.

To prove the statement (ii), consider the heterozygosity $h=1-\sum_{i=1}^{A_{0}}f_{i}^{2}$. The range of values of *h* is $0\leq h\leq 1-1/A_{0}$. There are segregating alleles in the population if and only if h>0. Its expected value E[h] satisfies the well-known relation

(16) $$\frac{dE[h]}{dt}=-\frac{E[h]}{N(t)}\;\;\Rightarrow \;\;\frac{dE[h]}{d\tau }=-\frac{E[h]}{N_{0}}$$

leading to an exponential decay of the expected heterozygosity

(17) $$E\left[h\left(t\right)\right]=h\left(0\right)e^{-\tau \left(t\right)/N_{0}}\;\;\Rightarrow \;\;\lim_{t\to \infty }E\left[h\left(t\right)\right]=h\left(0\right)e^{-\tau_{max}/N_{0}}$$

Since $h\leq (1-1/A_{0})$ and since h≠0⇔A(t)>1, the expected heterozygosity is bounded by $E\left[h\left(t\right)\right]\leq \left(1-1/A_{0}\right)P\left[A\left(t\right)>1\right]$. Hence, $c=\lim_{t\to \infty }P\left[A\left(t\right)>1\right]\geq h_{0}{\left(1-1/A_{0}\right)}^{-1}e^{-\tau_{max}/N_{0}}=p_{\infty }$. □

The second part of the theorem leads to this corollary.

**Corollary** **1.**  *If $\int_{0}^{\infty }dt/\lambda \left(t\right)<+\infty$, the expected times to fixation/loss are infinite:*

(18) $$E\left[t_{L}\right]=+\infty \;,\;E\left[t_{F}\right]=+\infty$$

For growing populations with $\tau_{max}<+\infty$, the expected values of $\tau_{L}$ and $\tau_{F}$ are always finite and satisfy the equations

(19) $$E\left[\tau_{L}\right]=2\frac{S(0)-E[s(\infty )]}{A_{0}-1}$$

(20) $$E\left[\tau_{F}\right]=2S_{C}\left(0\right)-2E\left[s_{C}\left(\infty \right)\right]$$

Since $\tau_{L},\tau_{F}\leq \tau_{max}$, we can obtain lower bounds on the final expected values of the rescaled entropy-like measures:

(21) $$E\left[s\left(\infty \right)\right]\geq 2S\left(0\right)-\left(A_{0}-1\right)\tau_{max}$$

(22) $$E\left[s_{C}\left(\infty \right)\right]\geq 2S_{C}\left(0\right)-\tau_{max}$$

## 3. Results

### 3.1. Constant Population Size

The neutral evolution of a biallelic variant implies a constant dissipation of allelic entropy in time:

(23) $$E\left[\Delta S|f\right]=-\frac{\delta_{\left[0<f<1\right]}}{2}$$

The rate of dissipation is independent on *S* or *f*, provided that the alleles are still segregating. Note that the only functions of *f* with this property are linear combinations of the entropy, the frequency *f* (which is a martingale under neutral evolution) and a constant.

We present an alternative derivation of the classical Formula (1) for the expected time to fixation based on the rate of entropy dissipation. Denote by $t_{FL}$ the time to fixation or loss of one of the alleles from initial frequencies $f_{0}$ and $1-f_{0}$. The time $t_{FL}$ is a random variable with probability density denoted by $p[t_{FL}=t]$. Note that

(24) $$P\left[t_{FL}>t\right]=P\left[0<f\left(t\right)<1\right]=E\left[\delta_{\left[0<f(t)<1\right]}\right],$$

since all terms are equivalent to the probability that the alleles are still segregating at time *t*. The expected time to fixation or loss is then

(25) $$\begin{array}{cc} E[t_{FL}]= & \int_{0}^{\infty }dt\;t\;p\left[t_{FL}=t\right]=\int_{0}^{\infty }dt\;P\left[t_{FL}>t\right]=\int_{0}^{\infty }dt\;E\left[\delta_{\left[0<f(t)<1\right]}\right]\\ = & -2\int_{0}^{\infty }dt\;E\left[\Delta S|f\left(t\right)\right]=-2E\left[S\left(f\left(\infty \right)\right)\right]+2S\left(f_{0}\right) \end{array}$$

where we used Equation (24) and the average of Equation (23) over the realizations of the process. Since this quantity is finite, the probability that the alleles are still segregating at t=∞ is zero, hence f(∞)∈{0,1} and the entropy S(f(∞))=0. This way, we obtain the final result

(26) $$E\left[t_{FL}\right]=2S\left(f_{0}\right)=-2N\left(f_{0}\ln\left(f_{0}\right)+\left(1-f_{0}\right)\ln(1-f_{0})\right)$$

This derivation shows that the relation between fixation time and entropy is a consequence of the linear decay of the average entropy of the system.

For multiple alleles, the decay in entropy does not depend on allele frequencies, but it is related only to the number of segregating alleles:

(27) $$E\left[\Delta S\right]=-\frac{A(t)-1}{2}$$

Interestingly, this implies that the initial entropy of the system is related to the average lifetime of a random allele doomed to extinction

(28) $$E\left[t_{L}\right]=\frac{2S_{0}}{A_{0}-1}=-\frac{2N}{A_{0}-1}\sum_{i=1}^{A_{0}}f_{i,0}\ln(f_{i,0})$$

For $A_{0}=2$, extinction of one of the two alleles means fixation of the other, hence this result reduces to Equation (26).

Note that the time until loss or fixation of a given allele (e.g., the *i*th allele) is instead

(29) $$E\left[t_{FL,i}\right]=-2N\left(f_{i,0}\ln(f_{i,0})+\left(1-f_{i,0}\right)\ln(1-f_{i,0})\right),$$

as derived by pooling all the other alleles together—which does not change their evolution in frequency, since they are interchangeable due to the neutrality—and using Equation (26).

The expected time to fixation of the last surviving allele is

(30) $$E\left[t_{F}\right]=-2N\sum_{i=1}^{A_{0}}\left(1-f_{i,0}\right)\ln(1-f_{i,0})$$

Note that despite the appearance, the right-hand side of this equation has no direct interpretation as an entropy. It is rather related to a difference in entropies conditioned on different macroscopic variables.

### 3.2. Variable Population Size

The previous results can be generalized to models with variable population size N(t) by a well-known time rescaling [7]. In fact, we find that the average decay of rescaled entropy is linear in the rescaled time $\tau =\int_{0}^{t}du/\lambda \left(u\right)$.

Therefore, for most demographic dynamics, the formulae of the previous sections retain their validity once each time is replaced by the corresponding rescaled time. For example, Equation (28) can be generalized for $E[\tau_{L}]$:

(31) $$E\left[\tau_{L}\right]=E\left[\int_{0}^{t_{L}}\frac{dt}{\lambda (t)}\right]=-\frac{2N_{0}}{A_{0}-1}\sum_{i=1}^{A_{0}}f_{i,0}\ln(f_{i,0})$$

and Equation (30) can be generalized for $E[\tau_{F}]$:

(32) $$E\left[\tau_{F}\right]=E\left[\int_{0}^{t_{F}}\frac{dt}{\lambda (t)}\right]=-2N_{0}\sum_{i=1}^{A_{0}}\left(1-f_{i,0}\right)\ln(1-f_{i,0})$$

However, this is not the case for populations that experience superlinear growth (for example, an exponential growth $N\left(t\right)/N_{0}=e^{\alpha t}$ with α>0). In this case, the genetic drift is effectively frozen after a finite time. This implies that there could be segregating alleles at arbitrarily long times with finite probability. This is actually what happens:

**Theorem** **1.**  *Consider a population with time-varying effective size, initially harboring a finite number of neutral alleles.*

*(i)*  *If $\tau_{max}=+\infty$, which includes populations growing linearly or less than linearly, then all alleles will either get fixed or disappear in finite time.*
*(ii)*  *If $\tau_{max}<+\infty$, which includes populations growing exponentially or faster, there is a nonzero probability that alleles will segregate forever in the population.*

The second part of the theorem leads to this corollary:

**Corollary** **1.**  *The expected times to fixation/loss for neutral alleles in exponentially growing populations (and other populations with $\tau_{max}<+\infty$) are infinite.*

This is true also for the relevant case of exponential growth. Denote the probability that alleles are segregating in the population at time *t* by P[A(t)>1]. For an exponentially growing population at rate α>0, the expected times to fixation/loss are infinite, and the probability of observing segregating alleles at time t=+∞ is at least

(33) $$P\left[A\left(\infty \right)>1\right]\geq \frac{h_{0}}{1-1/A_{0}}e^{-1/N\alpha }.$$

in terms of the initial number of alleles $A_{0}$ and heterozygosity $h_{0}$.

However, even for rapidly growing populations, the expected values of $\tau_{L}$ and $\tau_{F}$ are always finite and bounded by

(34) $$E\left[s\left(\infty \right)\right]\geq 2S\left(0\right)-\left(A_{0}-1\right)\tau_{max}$$

(35) $$E\left[s_{C}\left(\infty \right)\right]\geq 2S_{C}\left(0\right)-\tau_{max}$$

This suggests that populations experiencing a more extreme growth (i.e., smaller $\tau_{max}$) are guaranteed to maintain a higher fraction of the initial genetic diversity, as confirmed by the decay of heterozygosity $E\left[h_{\infty }\right]=h_{0}e^{-\tau_{max}/N}$.

In the simplest case of an exponential growth with rate α, we have that the final entropy satisfies the bounds

(36) $$E\left[s\left(\infty \right)\right]\geq 2S\left(0\right)-\left(A_{0}-1\right)/\alpha \;\;,\;\;E\left[s_{C}\left(\infty \right)\right]\geq 2S_{C}\left(0\right)-1/\alpha$$

## 4. Discussion

Our results show that entropy plays an unexpected role in the Kolmogorov equations describing the diffusion approximation to the Wright–Fisher model. The identification of the times until fixation and loss with the initial entropy of the system and related measures stems from this special role.

While in this work we connected the fixation times with the properties of allelic entropy and its dissipation under neutrality, it is not clear if these special properties arise by chance or if they are a consequence of more general relations between population genetics, partial differential equations, statistical mechanics and information theory. Entropy dissipation methods for the study of partial differential equations and nonlinear systems [8] do not consider the entropy of a random variable but rather the entropy of its distribution. For similar reasons, even mappings between population genetics and statistical mechanics (see e.g., Sella and Hirsh [9], Mustonen and Lässig [10]) do not seem to offer an immediate explanation of the linear decay of the entropy function.

For populations of variable size, entropy decay is time-dependent and generally nonlinear in time. In populations with linear or sublinear growth, expected fixation times are finite and related to entropy for populations through a nonlinear time transformation. On the other hand, superlinear growth (i.e., $\int_{0}^{\infty }dt/N\left(t\right)<+\infty$) is associated with infinite expected fixation times when the dynamics is neutral. These results open a series of questions on the interpretation of fixation and extinction probabilities and times in exponentially growing populations and the meaning of neutrality and positive selection. The theory of the fixation probabilities in growing populations [11] states that exponential expansion greatly increases the fixation probabilities of beneficial mutations. At the same time, our results prove that it strongly increases the lifetime of neutral polymorphism, reducing fixation probabilities. “Neutrality” here must be interpreted in a very strict sense, since in the long term the large population size would enhance any slightly beneficial or deleterious selective effect; in fact, the effective time-dependent population-scaled selection coefficient is $N_{e}\left(t\right)$ times the individual-level fitness effect. Any rapid unlimited growth is also an unrealistic demographic dynamics on long time scales; most natural populations experience exponential expansion only as a transient phase, e.g., colonization of a new niche or infection of a new host. The biological relevance of these asymptotic results should be revisited with an eye to the typical times of expansion and fixation in each case study.

## Data Availability

No new data were created.
